# High-throughput sequencing-based analysis of endogenetic fungal communities inhabiting the Chinese Cordyceps reveals unexpectedly high fungal diversity

**DOI:** 10.1038/srep33437

**Published:** 2016-09-14

**Authors:** Fei Xia, Xin Chen, Meng-Yuan Guo, Xiao-Hui Bai, Yan Liu, Guang-Rong Shen, Yu-Ling Li, Juan Lin, Xuan-Wei Zhou

**Affiliations:** 1 Key Laboratory of Urban Agriculture (South) Ministry of Agriculture, and Engineering Research Center of Cell & Therapeutic Antibody (Ministry of Education), and School of Agriculture and Biology, Shanghai Jiao Tong University, Shanghai 200240, People’s Republic of China; 2 State Key Laboratory of Genetic Engineering, School of Life Sciences, Fudan University, Shanghai 200438, People’s Republic of China; 3 Department of Immunology, University of Connecticut Health Center, Farmington, Connecticut 06032, United States; 4 Grassland Research Institute, Qinghai Academy of Animal Sciences and Veterinary Medicine, Qinghai Xining 810016, People’s Republic of China

## Abstract

Chinese Cordyceps, known in Chinese as “DongChong XiaCao”, is a parasitic complex of a fungus (*Ophiocordyceps sinensis*) and a caterpillar. The current study explored the endogenetic fungal communities inhabiting Chinese Cordyceps. Samples were collected from five different geographical regions of Qinghai and Tibet, and the nuclear ribosomal internal transcribed spacer-1 sequences from each sample were obtained using Illumina high-throughput sequencing. The results showed that Ascomycota was the dominant fungal phylum in Chinese Cordyceps and its soil microhabitat from different sampling regions. Among the Ascomycota, 65 genera were identified, and the abundant operational taxonomic units showed the strongest sequence similarity to *Ophiocordyceps, Verticillium, Pseudallescheria, Candida* and *Ilyonectria* Not surprisingly, the genus *Ophiocordyceps* was the largest among the fungal communities identified in the fruiting bodies and external mycelial cortices of Chinese Cordyceps. In addition, fungal communities in the soil microhabitats were clustered separately from the external mycelial cortices and fruiting bodies of Chinese Cordyceps from different sampling regions. There was no significant structural difference in the fungal communities between the fruiting bodies and external mycelial cortices of Chinese Cordyceps. This study revealed an unexpectedly high diversity of fungal communities inhabiting the Chinese Cordyceps and its microhabitats.

The biodiversity of fungi in taxonomy and biotopes produces a variety of secondary metabolites that display a broad range of bioactivities, from which important medicines have been discovered^1^. Studying the ecological factors underlying the dynamics of fungal communities remains a challenge because of the high taxonomic and ecological diversity^2^. Chinese Cordyceps (called “DongChong XiaCao” in Chinese phonetic alphabet), a unique species in the Qinghai-Tibet Plateau, is a parasitic complex of stromata and sclerotia formed with the parasitism of *Hepialus* spp. by *Ophiocordyceps sinensis*. Thoroughly permeated with mycelia, the dead larvae gradually grow into sclerotia, leaving only worm skins^3^. This phenomenon is similar to that of the parasitoidism observed in the insect kingdom^3^. Prior to 2007, the fungal strain *O. sinensis* was often confused with *Hirsutella sinensis* and Chinese Cordyceps, and it has been hypothesized that *Hirsutella sinensis* was the anamorph of Chinese Cordyceps. In addition, previous studies have miscalled the Chinese Cordyceps as *Ophiocordyceps sinensis*. Indeed, *Hirsutella sinensis* belongs to the genus *Ophiocordyceps* and family *Clavicipitaceae* according to a new classification principle and method^4^. In recent years, the mycobiota of Chinese Cordyceps has been extensively investigated using traditional culture-dependent methods. Approximately 600 isolates were obtained from different parts (including stromata, sclerotia, and external mycelial cortices) of Chinese Cordyceps and its soil microhabitats^5^. PCR-based molecular methods and sequencing of ribosomal DNA have been used to identify anamorphs of this species and investigate endogenetic fungal communities, and the results have provided insights into the ecological factors affecting the structure and diversity of endogenetic fungal communities^6^,^7^,^8^,^9^. In addition, many bioactive components have been isolated and identified from Chinese Cordyceps^10^, and more than 200 new bioactive metabolites have been isolated from the metabolites of endogenetic fungi (referred to as Cordyceps-colonizing fungi)^11^,^12^,^13^,^14^,^15^,^16^,^17^.

Fungal community analyses using large-scale sequencing techniques have been performed successfully for many years^18^. The nuclear ribosomal internal transcribed spacer (ITS) region is now widely used as a DNA molecular marker for the analysis of fungal communities using high-throughput sequencing^19^. With continuing improvements in sequencing techniques and the development of dedicated DNA databases, recent studies have demonstrated the potential of large-scale sequencing of ITS regions for quantifying and characterizing the fungal diversity in special biological samples, such as the pile-fermentation of puer tea^20^, plant endophytic fungi^21^ and rhizospheric fungi^22^. However, the diversity of the Chinese Cordyceps endogenetic fungal communities has not been evaluated using high-throughput sequencing.

Among the different high-throughput sequencing platforms, MiSeq has the highest throughput per run and lowest error rates^23^. Therefore, we used Illumina MiSeq sequencing to assess the fungal diversity in Chinese Cordyceps samples collected from five different geographical regions in the Tibet and Qinghai provinces. The abundance and diversity of the fungal communities from the Chinese Cordyceps samples were significantly higher than previously hypothesized. A few fungal taxa account for most of the species abundance, whereas the majority of species were only rarely retrieved. High-throughput sequencing will accelerate studies of the microbial diversity and community structure of Chinese Cordyceps and is beneficial for screening novel bioactive metabolites from different endogenetic fungal species.

## Results

### Alpha- diversity of endogenetic fungal community in Chinese Cordyceps

The alpha diversity of the endogenetic fungal communities in individual samples was explored using the Shannon-Weiner, Simpsons Diversity, Chao1 and Accumulated Cyclone Energy (ACE) indices (Table 1). Although the Shannon-Wiener index varied from 1.76 (in XM) to 3.92 (in ZS), the sequencing depth in each sample was sufficient for constituting the endogenetic fungal communities. The Shannon rarefaction plot showed that the diversity in the endogenetic fungal community never increased, according to the increased sequence amounts (Fig. S1). The soil samples had the largest fungal diversity compared with the fruiting bodies and external mycelial cortices, except for the QS and NyS samples. The Simpson index also showed a similar trend, consistent with the Shannon-Wiener index. However, the t-test showed no significant difference in the Shannon-Wiener index among fruiting bodies, external mycelial cortices and soil microhabitat samples of Chinese Cordyceps (*p* > 0.05). The Chao1 index and ACE estimator indicated that, in Chinese Cordyceps collected from Qumarlêb County, the richness of the fungal community was higher in the fruiting bodies of Chinese Cordyceps than in the external mycelial cortices and soil microhabitats. However, in the other four sampling areas, the fungal community richness was higher in the external mycelial cortices and soil microhabitats than in the fruiting bodies of Chinese Cordyceps. Nonetheless, the t-test of the Chao1 index and ACE estimator revealed no significant difference between the fruiting bodies, external mycelial cortices and soil microhabitats of Chinese Cordyceps in the five sampling areas (*p* > 0.05).

### Fungal community composition and soil microhabitats of Chinese Cordyceps

**Figure 1:**
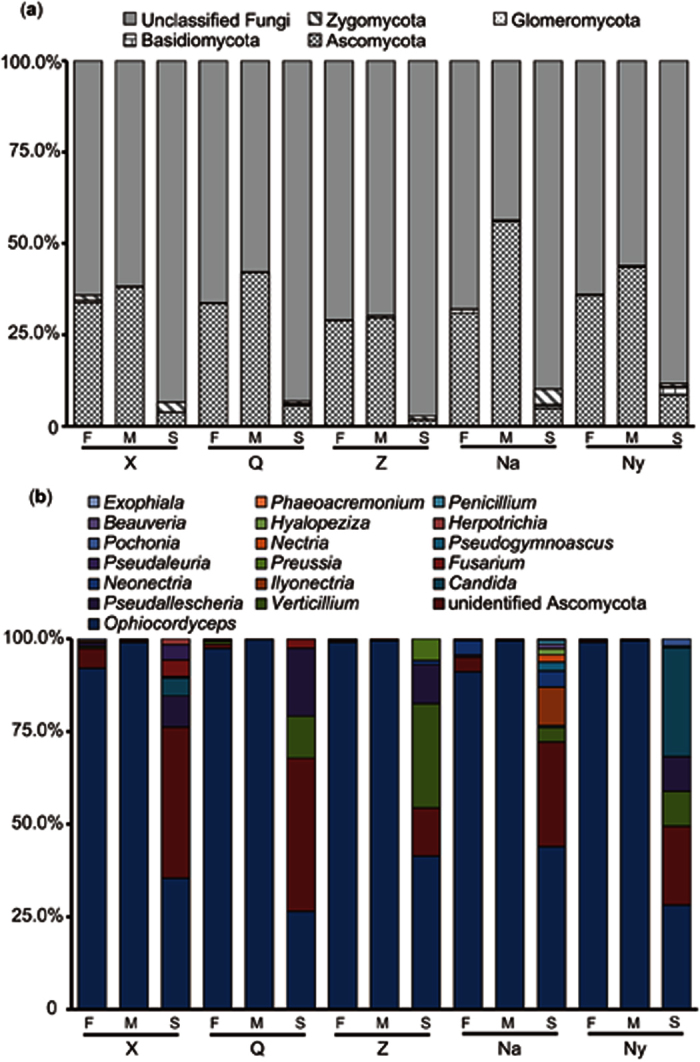
Endogenetic fungal community’s construction in Chinese Cordyceps and its microhabitat samples. (**a**) Endogenetic fungal communities at the phylum level; (**b**) Ascomycota endogenetic fungal communities at the genus level.

The fungal community composition and soil microhabitats of Chinese Cordyceps were estimated at the phylum and genus levels. Obviously, Ascomycota was the dominant fungus in the fruiting bodies of Chinese Cordyceps and external mycelial cortices from different sampling areas (Fig. 1a). A proportion of Ascomycota in the fruiting bodies of Chinese Cordyceps ranged from 28.95% (in ZF) to 35.84% (in NyF), which was higher in the external mycelial cortices samples, ranging from 29.64% (in ZM) to 56.01% (in NaF). However, the proportion of Ascomycota in the soil microhabitat was low, with 1.65% (in ZS) and 8.43% (in NyS). The proportions of Ascomycota in the fruiting bodies and external mycelial cortices of Chinese Cordyceps were significantly higher than in the soil microhabitats of the sampling areas (p < 0.01). However, the difference in the proportion of Ascomycota between the fruiting bodies and external mycelial cortices of Chinese Cordyceps was negligible (p = 0.09). In addition, a high proportion of unknown fungi were detected in the soil microhabitats of different sampling regions, in which the highest percentage was 97.26% in ZS, and the lowest percentage was 88.33% in NyS.

Moreover, the fungal types and compositions of Ascomycota were evaluated in the Chinese Cordyceps samples obtained from different sampling areas (Fig. 1b). Surprisingly, the genus *Ophiocordyceps* was overwhelmingly dominant in the fruiting bodies and external mycelial cortices of Chinese Cordyceps. The proportion of *Ophiocordyceps* in the Ascomycota phylum ranged from 90.57% (detected in NaF) to 99.14% (detected in ZF) in the fruiting bodies of Chinese Cordyceps. The proportion of *Ophiocordyceps* in the Ascomycota phylum was extremely high, accounting for more than 99% of all the samples of the external mycelial cortices. The t-test showed no significant difference in the proportions of *Ophiocordyceps* between the fruiting bodies and external mycelial cortices of Chinese Cordyceps (*p* > 0.05). However, the proportion of *Ophiocordyceps* in the Ascomycota phylum was much higher in the fruiting bodies and external mycelial cortices of Chinese Cordyceps than in the soil microhabitat samples (*p* < 0.01). In addition, similar amounts of other fungi were detected in the soil samples. The proportion of *Verticillium* in the Ascomycota phylum was 28.15% in ZS and 11.36% in QS. The proportion of *Pseudallescheria* in the Ascomycota phylum was 18.27% in QS and 10.50% in ZS. In addition, the phylum of Ascomycota, such as *Candida, Ilyonectria, Neonectria*, and *Fusarium*, were equally abundant in the soil microhabitat samples. As expected, a remarkable amount of unidentified Ascomycota was detected in the soil microhabitat samples, i.e., 41.30% in QS and 40.67% in XS.

### Comparison of endogenetic fungal communities

**Figure 2:**
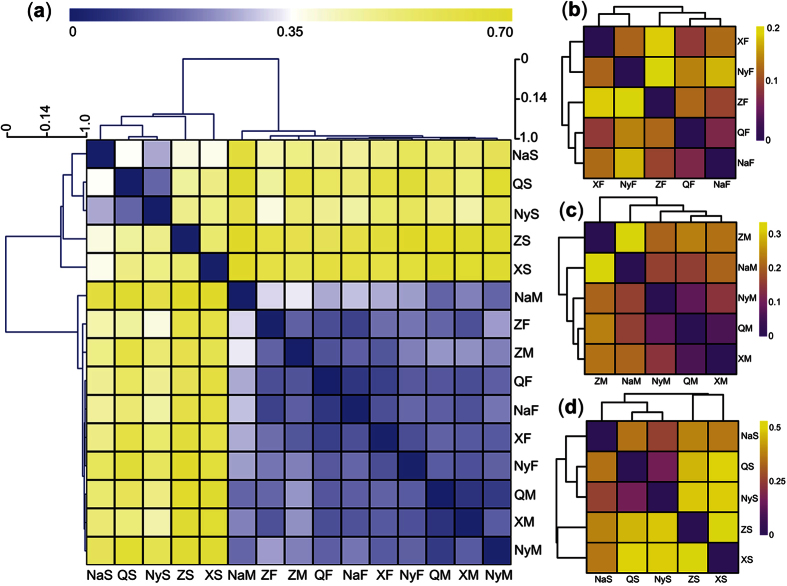
Hierarchical-clustering graphics of the weighted UniFrac pairwise distance between Chinese Cordyceps samples and the clustering dendrogram. (**a**) All of the samples of Chinese Cordyceps and its soil microhabitat samples; (**b**) fruiting bodies of Chinese Cordyceps; (**c**) external mycelial cortices of Chinese Cordyceps; (**d**) soil microhabitat samples collected from five different areas.

The fungal communities in the soil microhabitat samples were obviously different from the communities in the fruiting bodies and external mycelial cortices of Chinese Cordyceps (Fig. 2a). Hierarchical clustering of the weighted UniFrac distance between Chinese Cordyceps samples from different sampling areas indicated that the soil microhabitat samples did not cluster with the fruiting bodies and external mycelial cortices of Chinese Cordyceps (Fig. 2a). However, the fungal communities were similar between the fruiting bodies and external mycelial cortices of Chinese Cordyceps, as these samples clustered together despite the fact that they were obtained from different areas, indicating that certain fungal communities inhabit Chinese Cordyceps. In addition, the distance graph indicated that the fungal communities in the soil microhabitat samples were rather different from those in the fruiting bodies and external mycelial cortices of Chinese Cordyceps. The distance of the fungal communities between the soil microhabitat samples, fruiting bodies and external mycelial cortices of Chinese Cordyceps collected from different areas is shown in Table S1. Furthermore, we compared the soil microhabitat, fruiting body and external mycelial cortices samples collected from different sampling areas, respectively. The endogenetic fungal communities were different between fruiting bodies collected from the five areas. However, the distance values were not more than 0.2 (Table S1), which was quite small compared with the fungal community in the soil microhabitats (Fig. 2b). There was a difference between the endogenetic fungal communities in ZM and NaM (Fig. 2c), and the distance value was 0.34 (Table S1). However, a major dissimilarity was observed among the fungal communities in the soil microhabitat samples collected from different areas (Fig. 2d), indicating that the fungal communities were highly diverse in soil from different sampling areas.

**Figure 3:**
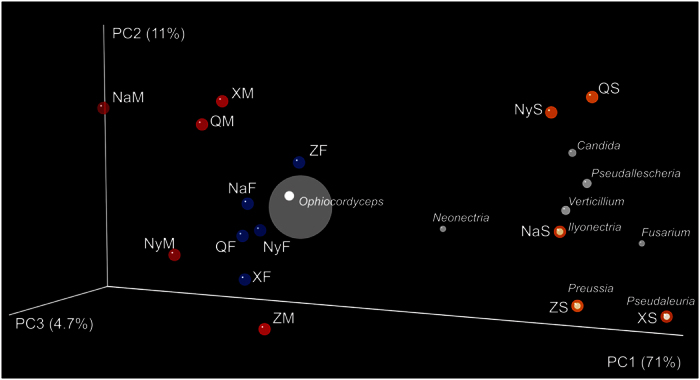
Principal coordinates analysis of endogenetic fungal communities in samples Chinese Cordyceps and soil microhabitat samples. Percentages on the axes of the graph are the explained variance of total variance. The OUT matrix used in the analyses was clustered at the 97% similarity and the principal coordinate’s analysis was based on Weighted UniFrac distances. The solid points in abbreviations of the sample name indicate the samples distributed in the ordination.

The results of the Weighted-UniFrac principal coordinates analysis also supported the clustering relationships between Chinese Cordyceps and soil microhabitat samples from different sampling areas (Fig. 3). Samples of the fruiting bodies and external mycelial cortices of Chinese Cordyceps clustered together, and the soil microhabitat samples separately clustered with themselves, consistent with the clustering dendrogram obtained from the Hierarchical-clustering analysis (Fig. 2a). In addition, the genus *Ophiocordyceps* primarily contributed to the cluster relationship between the fruiting bodies and external mycelial cortices of Chinese Cordyceps. However, as fungal communities were diverse in the soil microhabitat samples, the genus effect on the clustering relationship of the soil samples did not reflect a certain group of fungi.

### Abundance of endogenetic fungal communities

**Figure 4:**
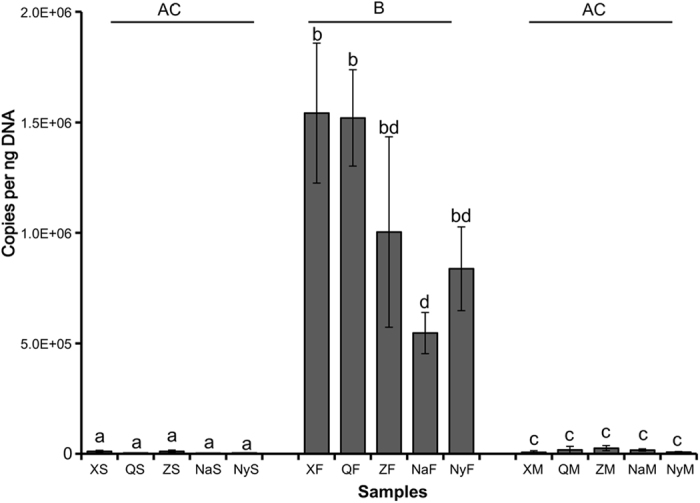
Copy numbers of ITS sequence in Chinese Cordyceps and its microhabitat samples. The error bars indicate SDs (n = 3). Different letters indicate significant differences (p < 0.05) of the ITS copies between the samples or the groups.

The copy number of ITS sequences per nano-gram (ng) genomic DNA represents the abundance of the fungal communities in the samples. The copy number of ITS sequences in the fruiting bodies of Chinese Cordyceps ranged from 5.47 ± 0.93 × 10^5^ (in NaF) to 1.54 ± 0.32 × 10^6^ (in XF) copies per ng DNA. However, the ITS copy number in the external mycelial cortices and soil microhabitats only ranged from 3.33 ± 1.02 × 10^3^ (in NaS) to 2.57 ± 1.17 × 10^4^ (in ZM) copies per ng DNA. The one-way ANOVA analysis indicated that the copy number in the fruiting bodies of Chinese Cordyceps was significantly higher than that in the external mycelial cortices and soil microhabitats (p < 0.05) (Fig. 4). However, there was no significant difference in the copy numbers between the external mycelial cortices and soil microhabitats. When considering the differences in the same samples from Chinese Cordyceps at different locations, there was no significant difference in ITS sequence copy numbers between samples of the soil microhabitats and external mycelial cortices. However, in the fruiting body samples, the ITS sequence copy number was a notable lower (*p* < 0.05) in NaF (Fig. 4).

## Discussion

**Figure 5:**
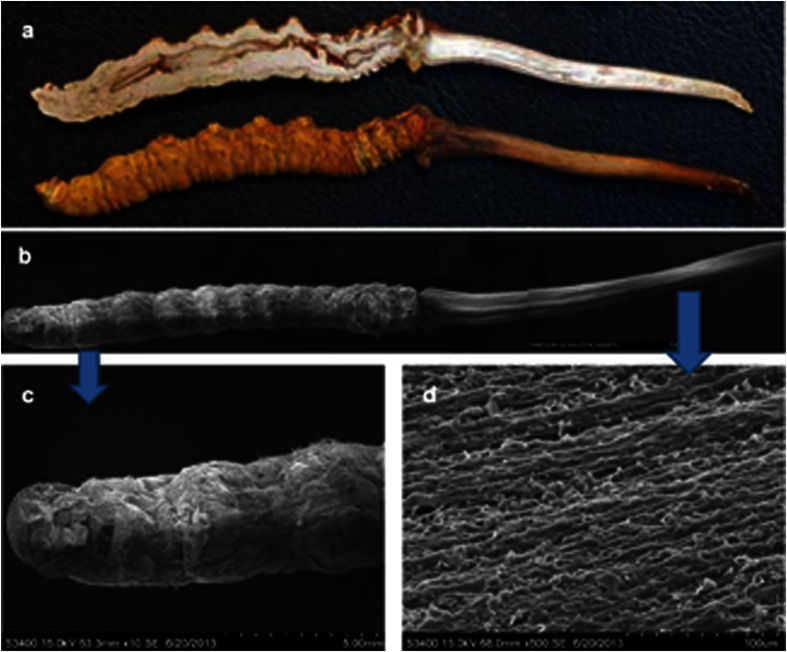
Scanning electron micrograph of mycelia covering the outside of a fruiting bodies of natural Chinese Cordyceps. (**a**) Anatomic structure of Chinese Cordyceps clearly showed the worm gut; (**b**) The spliced image of the Chinese Cordyceps segmentation using scanning electron microscopy (10×); (**c**) The sclerotia segmentation image shows that the lava was covered with mycelial cortices (10×); (**d**) The stromata segmentation image shows the fungal filaments on the surface of stromata (500×).

**Figure 6:**
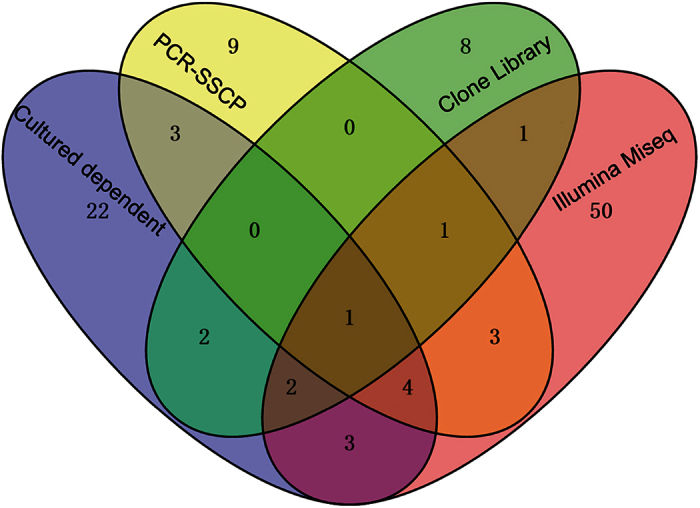
Comparison of the endogenetic fungal communities investigated using different methods. Different colors revealed the results in the current study and previously published studies using other methods for the analysis of the diversity of the endogenetic fungal communities, i.e., culture-dependent method^5^, PCR-SSCP^26^ and clone library^9^. The numbers indicate the shared genera in the studies.

As a mysterious material for traditional Chinese medicine, endogenetic fungal communities have been studied by many researchers, and many fungi have been isolated from Chinese Cordyceps using culture-dependent methods. Throughout the 1980s and until the end of the twentieth century, 22 fungi, spanning 13 genera associated with the anamorph of Chinese Cordyceps, were isolated^24^. Among these isolated fungi, novel fungal species, such as *Chrysosporium sinense* (Z.Q. Liang), *Mortierella hepiali* (Q.T. Chen & B. Liu), *Scytalidium hepiali* (C.L. Li), *Chrysosporium sinense* (Z.Q. Liang) and *Tolypocladium sinense* (C.L. Li), have been identified. Subsequently, the biodiversity of endogenetic fungal communities inhabiting the Chinese Cordyceps has gradually gained more attention. In 2010, 572 isolates were obtained from the Chinese Cordyceps and assigned to 37 genera according to morphological characteristics under culture conditions^5^. These studies, conducted based on the culture-dependent methods, preliminarily indicated the rich diversity of the endogenetic fungal communities inhabiting the Chinese Cordyceps. However, although numerous fungal isolates were identified, the endogenetic fungal communities in the Chinese Cordyceps were much more complex than initially considered. Scanning electron microscopy (Hitachi S-3400N, Hitachi High Technologies, Japan) revealed that the surface of the fruiting bodies of Chinese Cordyceps are covered with a mycelia complex, indicating an abundant community of fungi on the surface of the fruiting bodies of Chinese Cordyceps (Fig. 5). However, because of the limitation of the available techniques, the diversity and the structure of the endogenetic fungal communities of Chinese Cordyceps remains unclear. In the present study, 787,002 high-quality reads from 65 genera were identified from the Chinese Cordyceps using Illumina Miseq sequencing (Table S2). Compared with previous data, the Illumina Miseq sequencing analysis of the Chinese Cordyceps revealed a high degree of fungal diversity, much higher than the previous understanding. For example, Zhang *et al*.^5^ reported that 37 fungal genera were isolated from Chinese Cordyceps, however, only 10 genera were under investigation. The 55 fungal genera identified in the present study were not observed using culture-dependent methods (Fig. 6). The comparison of different methods demonstrated that only a small percentage of microorganisms can be cultivated using traditional cultivation techniques^25^.

With the development of molecular biological techniques, PCR-based methods were also employed to detect the endogenetic fungal communities of the Chinese Cordyceps. For example, Zhang *et al*.^26^ used the PCR-single-strand conformation polymorphism (PCR-SSCP) method to investigate endogenetic fungal communities, and only 266 clones were selected for sequencing and assigned to 21 genera^26^. Compared with the results obtained in the present study, 56 fungal genera were not detected. Xia *et al*.^9^ used the clone library method to analyze the composition of the endogenetic fungal communities inhabiting the Chinese Cordyceps, and 17 genera of fungi were detected in the larva and stromata of Chinese Cordyceps. Compared with the data obtained in the present study, the results obtained using the clone library method were rather limited. However, two fungal genera, *Cryptococcus* and *Mortierella*, were detected using PCR-SSCP and clone library methods, indicating the importance of these genera in the Chinese Cordyceps. Some species in the genus *Cryptococcus* (i.e., *C. neoformans* and *C. gattii.)* are important fungal pathogens of immunocompromised individuals^27^ and are likely involved during fungal infection in the development of the Chinese Cordyceps. *Mortierella hepiali* has been implicated in the formation of metabolites similar to the Chinese Cordyceps^28^,^29^. In the present study, the Illumina Miseq sequencing analysis of the fungal communities inhabiting the Chinese Cordyceps and its soil microhabitat samples revealed an unexpectedly high fungal diversity. The results also showed that the Chinese Cordyceps is a niche predominantly occupied by various fungi^26^,^30^,^31^. Without consideration of the undefined sequences, Ascomycota was the main phylum of fungal communities in the diverse samples and in samples obtained from different sampling regions. This result was consistent with previous reports because this phyla of fungi was widely distributed throughout the environment^2^, larvae gut^32^ and plant roots^33^.

In addition, there was an obvious difference in the fungal communities between the soil microhabitat samples and the Chinese Cordyceps. The microenvironment could influence the organism community. The soil microhabitat samples were remarkably diverse compared with the Chinese Cordyceps and could differently affect the organism communities. Previous studies have demonstrated that many factors, including the level of oxygen, moisture, pH and nutrient substance, could affect the microbial community^34^,^35^,^36^. The diversity of the endogenetic fungal communities inhabiting the fruiting bodies derived from the soil microhabitat of the Chinese Cordyceps and the diversity of fungal communities in the soil microhabitats varied among the samples obtained from separate sampling localities.

In previous studies, many scientists, particularly in China, have made great efforts to isolate fungi from the Chinese Cordyceps to identify its anamorph^7^,^37^,^38^,^39^,^40^. At least 13 genera, containing 30 fungal species, have previously been associated with the anamorph of the Chinese Cordyceps^24^. Recently, based on the opinion of “one fungus one name”^41^, increasing evidence has indicated that *O. sinensis* might be the anamorph of Chinese Cordyceps, but *C. multiaxialis* and *C. nepalensis* shared almost identical ITS sequences with *O. sinensis*^7^,^8^. However, only one study reported that the successful cultivation of Chinese Cordyceps under artificial conditions, and the components of the asexual state of the fungus were detected and analyzed using high performance liquid chromatography (HPLC) and ^1^H-NMR (^1^H-nuclear magnetic resonance) fingerprinting^3^. The mechanism underlying the fungal infection of an insect larva to form a Chinese Cordyceps with mature perithecium remains unclear^30^. The results of the high-throughput sequencing analysis showed that *Ophiocordyceps* was an overwhelmingly dominant fungus in Chinese Cordyceps fruit bodies and microhabitat, including the external mycelial cortices covering the larvae and the soil microhabitat. Together with previous studies using culture-dependent methods, these results further indicated that *Ophiocordyceps* was the nerveless sexual stage of the Chinese Cordyceps. These results were also consistent with the hypothesis that *H. sinensis* was also the nerveless sexual stage of the Chinese Cordyceps^8^,^42^. Therefore, the fungal strain *O. sinensis* should be same as *H. sinensis* reported in a previous study, consistent with the current opinion of most scientists in the field^41^. In addition, determining optimal fungal culture conditions for *Ophiocordyceps* fungi for future vaccination and isolation experiments, according to the Koch’s postulation, is urgently needed.

Clarification of the endogenetic fungal communities in the Chinese Cordyceps might contribute to the discovery of compounds with similar pharmaceutical functions in the Chinese Cordyceps and the identification of novel enzymes or compounds. We have demonstrated that some fungal species isolated from Chinese Cordyceps produce the same or similar medicinal components as Chinese Cordyceps^43^. Some novel compounds were discovered while analyzing the secondary metabolites of fungi isolated from the Chinese Cordyceps, and these novel compounds demonstrated antibacterial, antitumor and anti-HIV potential^43^. Using high-throughput sequencing to explore the endogenetic fungal communities in the Chinese Cordyceps could not only establish a guideline for isolating fungi that produce functional enzymes and compounds but also contribute to the discovery of novel functional genes for the production of new proteins using engineering techniques.

We described fungal communities inhabiting the Chinese Cordyceps and microenvironment, some of which had been reported in previous studies. We inferred that Ascomycota was the dominant fungus phylum in fruiting bodies and external mycelial cortices of Chinese Cordyceps and soil microhabitats in different sampling areas. We also identified 65 genera, and the most abundant genera were *Ophiocordyceps, Verticillium, Pseudallescheria, Candida* and *Ilyonectria Ophiocordyceps* was overwhelmingly dominant in the fruiting bodies and external mycelial cortices of the Chinese Cordyceps. However, the fungal community in soil microhabitat samples was more diverse than that on the Chinese Cordyceps. Weighted UniFrac distance indicated that the fungal communities in soil microhabitats were not clustered with those in the external mycelial cortices and fruiting bodies of the Chinese Cordyceps. The fungal communities from fruiting bodies of the Chinese Cordyceps and mycelial membrane were similar in different sampling areas. However, significant dissimilarity was observed among the fungal community in the soil microhabitat samples collected in different areas.

## Material and Methods

### Sampling and sample processing

**Figure 7:**
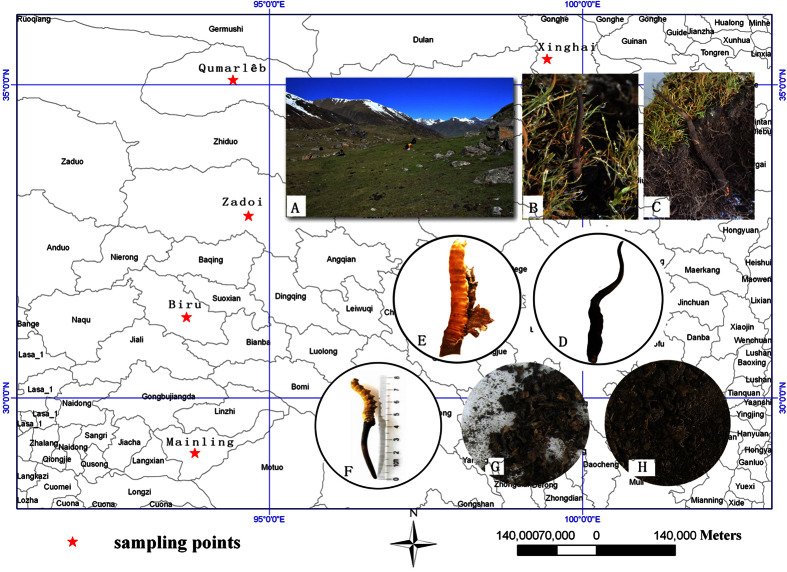
Sampling and sample processing. “★” collecting site of the Chinese Cordyceps, including three counties of Qinghai, and two counties of Tibet, the maps in the figure were generated using ArcGIS 10.0 software. (**A**) Plateau meadow; (**B,C**) growing Chinese Cordyceps collected during early fruiting stage (prior to the stromata production of spores); (**D**) early harvesting sample; (**E**) splitting the mycoderma and sclerotium; (**F**) fruiting bodies of the Chinese Cordyceps (abbreviated as “F”); (**G**) sample of external mycelial cortices (abbreviated as “M”) and (**H**) sample of soil (abbreviated as “S”) isolated from of the membrane covering the Chinese Cordyceps. For sample collection sites, the counties of Xinghai, Qumarlêb County and Zadoi of Qinghai province were abbreviated as “X”, “Q” and “Z”, respectively. Biru county of Nagqu and Mainling County of Nyingchi City of the Tibet Autonomous Region were abbreviated as “Na” and “Ny”, respectively.

As the types of Chinese Cordyceps vary in different areas, Chinese Cordyceps samples were collected from five counties in different dimensions, including Xinghai, Qumarlêb and Zadoi in Qinghai province and Biru and Mainling in the Tibet Autonomous Region (Fig. 7). Chinese Cordyceps samples were collected during early fruiting stages of *O. sinensis* (i.e., prior to sporangium formation and stromata spore production)^44^. As the mature stage of Chinese Cordyceps is earlier in Qinghai than in Tibet, the fruiting stage period occurred from April to May of 2015 in Qinghai and May to June of 2015 in Tibet. At least 30 samples were collected from three random plots at 100 meters apart, and 5–10 samples were collected from each plot in a random manner. The collected samples were stored in sterile plastic bags and carried to the laboratory of Plant Biotechnology R&D Center of Shanghai Jiao Tong University in ice boxes.

All samples were divided into three groups: (1) fruiting bodies including stromata and sclerotia; (2) microhabitat including external mycelial cortices that cover larvae; and (3) the soil adhering to the surface of the membrane covering Chinese Cordyceps. The names of the three groups were abbreviated as “F”, “M”, and “S”, respectively (Fig. 7F–H). The five sampling locations, including Xinghai, Qumarlêb, Zadoi of Qinghai province, and Biru, Mainling of the Tibet Autonomous Region, were abbreviated as “X”, “Q”, “Z”, “Na” and “Ny”, respectively. For later discussion, we refer to the samples according to the abbreviations of the sampling locations tracking the abbreviation of sample group names. For example, sample “XF” indicates that the sample contains fruiting bodies gathered from Xinghai County. The fruiting bodies were sterilized using 75% ethanol for 2–3 minutes, followed by 2.5% sodium hypochlorite for 20–25 minutes. Subsequently, the samples were rinsed three times with sterile water^9^ and stored at −20 °C until the genomic DNA was extracted for further endogenetic fungal community analysis.

### DNA extraction, PCR amplification and high-throughput sequencing

Prior to DNA extraction, the samples were ground in liquid nitrogen to improve the efficiency of the fungal DNA extraction. Subsequently, the total genomic DNA from each sample was isolated using the PowerSoil^TM^ soil DNA Isolation Kit (Mo Bio Laboratories, Solana Beach, CA, USA) according to the manufacturer’s instructions. The ITS1 region sequence was used for the endogenetic fungal community analysis with primer set NSI1 (GATTGAATGGCTTAGTGAGG) and 58A2R (CTGCGTTCTTCATCGAT)^45^. To distinguish between each sample, a unique barcode containing 12 nucleotides was added in the forward primer for each sample. The PCR amplification reaction was conducted as previously described^9^. A 5-minute initial denaturation step at 95 °C was followed by 30 cycles in the following conditions: 30 seconds at 94 °C, 30 seconds at 52 °C, 1 min at 72 °C. A final 10 minute elongation step at 72 °C completed the thermal program. The amplifications were conducted in triplicate and pooled to minimize the PCR bias. Subsequently, the PCR products were identified using electrophoresis on a 1.5% agarose gel, and the appropriate fragments were purified using the DNA Gel Extraction Kit (Axygen, Union City, CA, USA). After the concentration of the PCR products was assayed using a Qubit^®^2.0 Fluorometer and the Qubit dsDNA HS Assay kit (Life Technologies, Invitrogen division, Darmstadt, Germany), the purified PCR amplicons were equimolarly pooled and subsequently used for library construction and sequencing on a Miseq PE250 platform (Illumina, USA) according to the instructions of the Shanghai Genenergy Biotechnology Co. Ltd.

### Quantitative PCR

SYBR Green I-based quantitative PCR was employed to detect the copy numbers of the ITS sequences in the DNA of the samples obtained from different locations. The primer set NSI1 and 58A2R^45^ was same as that used in the high-throughput sequencing. The qPCR reaction mix and the thermal program were the same as previously described^9^, with the same annealing temperature as that used in for high-throughput sequencing PCR. qPCR was conducted in triplicate for each sample. The data were analyzed using MxPro qPCR software version 3.0 (Stratagene, USA). Standard curves had R^2^ > 0.99 and the efficiencies for PCR reactions were 102.2% for the fungal community. The copy numbers of the ITS sequences between different samples were examined using one-way ANOVA analysis in Statistical Product and Service Solutions software (SPSS, version 19.0 for Windows, SPSS Inc., Chicago, IL, USA). A P value < 0.05 was considered statistically significant.

### Sequence analysis

The sequence data were processed using QIIME, version 1.7.0^46^. Briefly, each sample sequence was highlighted based on the 12-nucletide (nt) barcodes with the criteria of average quality value higher than 25 and sequence length of more than 200 bp. After the high-quality read pairs were merged into single contigs using “join paired ends.py” script in QIIME, 787,002 high-quality reads were obtained in total, with at least 13,855 reads in each sample. The merged reads were chimera-checked using USEARCH61^47^ against the database of UNITE^48^. Subsequently, the denoised reads were clustered into operational taxonomic units (OTUs) according to the pipeline of QIIME commands (http://qiime.org/genindex.html) based on 97% identity. Representative sequences of each OUT were blasted against the UNITE database to obtain taxonomy with a minimum confidence of 80%. Alpha diversity of fungal communities in each sample was represented using Shannon-Weiner, Chao1, ACE and Simpson indices and was calculated after the reads were normalized to the minimum reads (13855 reads) in each sample. Euclidean-based Weighted UniFrac distances^49^ were employed to determine the distance (beta diversity) between fungal communities in any pair of samples. The hierarchical clustering graph was generated using the MeV software, version 4.9.0^50^ and the HCL-Hierarchical clustering method^51^. The principal coordinates analysis (PCoA) was performed based on the Weighted UniFrac distance matrices using the command of “beta diversity through plots.py” and visualized using KiNG Display software (http://kinemage.biochem.duke.edu/software/king.php).

## Additional Information

**Accession codes**: The sequence data obtained in the current study were submitted to the NCBI GenBank Short Read Archive (SRA) under accession number SRP067896.

**How to cite this article**: Xia, F. *et al*. High-throughput sequencing-based analysis of endogenetic fungal communities inhabiting the Chinese Cordyceps reveals unexpectedly high fungal diversity. *Sci. Rep.* **6**, 33437; doi: 10.1038/srep33437 (2016).

## Supplementary Material

Supplementary Information

## Figures and Tables

**Table 1:** Alpha- diversity indexes of fungal community in different samples of Chinese Cordyceps collected in different areas.

	Shannon			Simpson			Chao			ACE		
	F^b^	M	S	F	M	S	F	M	S	F	M	S
Q^a^	3.05^c^	1.95	2.28	0.14	0.26	0.19	313.80	314.11	239.00	311.47	304.61	250.98
X	3.13	1.76	3.33	0.12	0.28	0.11	253.71	280.23	283.56	254.72	270.10	285.67
Z	2.46	3.13	3.92	0.18	0.11	0.04	253.64	319.28	311.12	252.80	317.44	308.32
Na	2.72	2.01	3.62	0.15	0.33	0.07	252.72	268.71	328.00	257.49	287.15	318.85
Ny	2.13	2.56	1.93	0.21	0.21	0.36	284.78	311.44	298.10	275.16	312.35	296.44

^a^Abbreviation of the sampling sites. The Xinghai, Qumarlêb and Zadoi counties of Qinghai province were abbreviated as “X”, “Q” and “Z”, respectively. Biru county of Nagqu and Mainling County of Nyingchi City of the Tibet Autonomous Region were abbreviated as “Na” and “Ny”, respectively.

^b^Abbreviation of different samples of Chinese Cordyceps. Fruiting bodies of Chinese Cordyceps, external mycelial cortices and soil adhere to Chinese Cordyceps were abbreviated as “F”, “M” and “S”, respectively.

^c^All the indices were calculated after the reads were normalized to minimum (13,855 reads) in each sample.
