# Supplementary material for: High-throughput sequencing-based analysis of endogenetic fungal communities inhabiting the Chinese Cordyceps reveals unexpectedly high fungal diversity
Source: Sci Rep. 2016 Sep 14;6:33437. doi: 10.1038/srep33437 (PMC5022047; doi:10.1038/srep33437)
Supplement: Supplementary Information [file srep33437-s1.pdf]

Supplemental information:

Research article

High-throughput sequencing-based analysis of endogenetic fungal communities inhabiting the Chinese Cordyceps reveals unexpectedly high fungal diversity

Fei Xia[1, 2], Xin Chen[3], Meng-Yuan Guo[1], Xiao-Hui Bai[1], Yan Liu[1], Guang-Rong Shen[1], Yu-Ling Li[4], Juan Lin[2*], Xuan-Wei Zhou[1*]

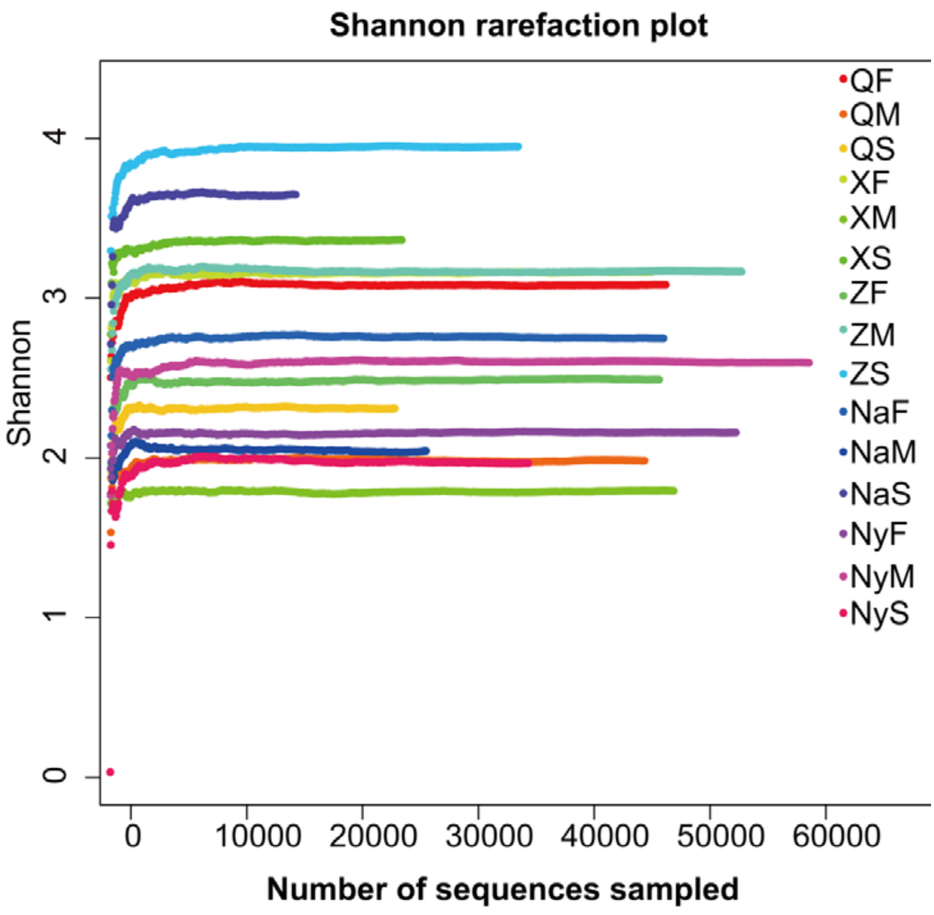

Fig. S1 Rarefaction curve of the ITS sequences. In the panel, the x-axis represents the number of sequences and the y-axis represents the number of operational taxonomic units (OTUs) determined at a 97% similarity.

Table S1 Distance between fungal endophyte communities inhabiting different samples of natural DCXC collected from different areas

	XF	XM	XS	QF	QM	QS	ZF	ZM	ZS	NaF	NaM	NaS	NyF	NyM	NyS
XO	0														
XM	0.14	0													
XS	0.62	0.68	0												
QO	0.09	0.12	0.63	0											
QM	0.16	0.07	0.69	0.14	0										
QS	0.62	0.51	0.5	0.56	0.58	0									
ZO	0.18	0.13	0.62	0.12	0.17	0.47	0								
ZM	0.13	0.23	0.59	0.13	0.24	0.62	0.16	0							
ZS	0.64	0.69	0.53	0.58	0.7	0.46	0.62	0.60	0						
NaO	0.12	0.13	0.63	0.07	0.15	0.52	0.10	0.15	0.62	0					
NaM	0.26	0.26	0.75	0.26	0.16	0.68	0.32	0.34	0.76	0.29	0				
NaS	0.50	0.53	0.37	0.48	0.53	0.35	0.44	0.49	0.39	0.47	0.64	0			
NyO	0.12	0.13	0.65	0.14	0.14	0.66	0.19	0.21	0.75	0.17	0.25	0.57	0		
NyM	0.14	0.14	0.68	0.15	0.08	0.68	0.25	0.21	0.69	0.19	0.16	0.58	0.15	0	
NyS	0.54	0.45	0.49	0.48	0.50	0.16	0.39	0.53	0.49	0.45	0.61	0.26	0.56	0.59	0

Table S2. Summary of detected fungus at each taxonomy level

No.[a]	**Phylum**[b]	**Class**	**Order**	**Family**	**Genus**	**Species**
1	Ascomycota	Agaricomycetes	Agaricales	Amanitaceae	*Alatospora*	*Alatospora* acuminata
2	Basidiomycota	Agaricostilbomycetes	Agaricostilbales	Ascobolaceae	*Amanita*	*Apodus* sp.
3	Glomeromycota	Dothideomycetes	Auriculariales	Bionectriaceae	*Apodus*	*Ascobolaceae* sp.
4	Zygomycota	Eurotiomycetes	Cantharellales	Ceratobasidiaceae	*Ascobolus*	*Ascobolus* sp.
5	Unidentified	Glomeromycetes	Capnodiales	Chaetomiaceae	*Aspergillus*	*Ascomycota* sp. FL-2010c
6		Incertae_sedis	Chaetosphaeriales	Chaetosphaeriaceae	*Beauveria*	*Candida* catenulata
7		Leotiomycetes	Chaetothyriales	Clavariaceae	*Candida*	*Candida* glaebosa
8		Microbotryomycetes	Coniochaetales	Clavicipitaceae	*Cephaliophora*	*Cephaliophora* sp.
9		Pezizomycetes	Cystofilobasidiales	Cordycipitaceae	*Chaetomium*	*Cladosporium* halotolerans
10		Saccharomycetes	Diaporthales	Cortinariaceae	*Cladophialophora*	*Clavaria* californica
11		Sordariomycetes	Eurotiales	Cucurbitariaceae	*Cladosporium*	*Clavaria* sp.
12		Tremellomycetes	Filobasidiales	Cystofilobasidiaceae	*Clavaria*	*Clonostachys* rosea f. catenulata
13		Ustilaginomycetes	Glomerales	Davidiellaceae	*Clonostachys*	*Coniochaetales* sp.
14		Unidentified	Helotiales	Filobasidiaceae	*Cortinarius*	*Cryptococcus* aerius
15			Hypocreales	Glomeraceae	*Cryptococcus*	*Cylindrotrichum* sp.

16	Incertae_sedis	Glomosporiaceae	*Curvularia*	*Exophiala* salmonis
17	Leotiales	Herpotrichiellaceae	*Cylindrotrichum*	*Filobasidium* uniguttulatum
18	Leucosporidiales	Hyaloscyphaceae	*Exophiala*	*Fontanospora* eccentrica
19	Malasseziales	Hygrophoraceae	*Filobasidium*	*Funneliformis* mosseae
20	Microascales	Incertae_sedis	*Fontanospora*	*Fusarium* brachygibbosum
21	Mortierellales	Inocybaceae	*Funneliformis*	*Fusarium* cf dimerum 21535
22	Pezizales	Lasiosphaeriaceae	*Fusarium*	*Fusarium* pseudensiforme
23	Pleosporales	Leotiaceae	*Glarea*	*Guehomyces* pullulans
24	Russulales	Leucosporidiaceae	*Guehomyces*	*Herpotrichia* coulteri
25	Saccharomycetales	Massarinaceae	*Herpotrichia*	*Hyalopeziza* sp. TNS-F17975
26	Sebacinales	Microascaceae	*Hyalopeziza*	*Hygrocybe* conica
27	Sordariales	Mortierellaceae	*Hygrocybe*	*Ilyonectria* mors-panacis
28	Sporidiobolales	Mycenaceae	*Ilyonectria*	*Inocybe* ericetorum
29	Thelephorales	Nectriaceae	*Inocybe*	*Malassezia* restricta
30	Tremellales	Ophiocordycipitaceae	*Lachnum*	*Mastigobasidium* sp.
31	Trichosporonales	Plectosphaerellaceae	*Lecanicillium*	*Metarhizium* pinghaense
32	Urocystidales	Pleosporaceae	*Malassezia*	*Mortierella* acrotona

33	Xylariales	Pseudeurotiaceae	*Mastigobasidium*	*Mortierella* amoeboidea
34	Unidentified	Pyronemataceae	*Metarhizium*	*Mortierella* bainieri
35		Russulaceae	*Mortierella*	*Mortierella* elongatula
36		Saccharomycetaceae	*Mrakia*	*Mortierella* pseudozygospora
37		Schizophyllaceae	*Mycena*	*Mortierella* sp. 04M 158
38		Sclerotiniaceae	*Myrothecium*	*Mortierella* sp. WD32A
39		Sebacinaceae	*Nectria*	*Mrakia* frigida
40		Sebacinales_Group_B	*Neonectria*	*Mycena* cinerella
41		Sporormiaceae	*Ophiocordyceps*	*Myrothecium* tongaense
42		Teratosphaeriaceae	*Penicillium*	*Nectria* ramulariae
43		Thelephoraceae	*Phaeoacremonium*	*Ophiocordyceps* sinensis
44		Togniniaceae	*Phialocephala*	*Penicillium* bialowiezense
45		Trichocomaceae	*Pochonia*	*Phaeoacremonium* tuscanum
46		Trichosporonaceae	*Preussia*	*Phialocephala* fortinii
47		Vibrisseaceae	*Pseudaleuria*	*Pleosporaceae* sp. RS-5
48		Unidentified	*Pseudallescheria*	*Pochonia* sp.
49			*Pseudogymnoascus*	*Preussia* terricola

50	*Pyrenochaetopsis*	*Pseudaleuria* sp.
51	*Russula*	*Pseudallescheria* fimeti
52	*Saccharicola*	*Pseudogymnoascus* roseus
53	*Schizophyllum*	*Russula* citrinochlora
54	*Sebacina*	*Russula* sp. OTU322
55	*Serendipita*	*Saccharicol*a sp.
56	*Sporobolomyces*	*Sebacina* mycobiont
57	*Stachybotrys*	*Sebacina* incrustans
58	*Sterigmatomyces*	*Serendipita* herbamans
59	*Tetracladium*	*Slopeiomyces* cylindrosporus
60	*Thecaphora*	*Sporobolomyces* coprosmae
61	*Tomentella*	*Stachybotrys* oleronensis
62	*Trichosporon*	*Sterigmatomyces* halophilus
63	*Verticillium*	*Thecaphora* amaranthi
64	*Volutella*	*Trichosporon* dehoogii
65	*Zygosaccharomyces*	*Verticillium* dahliae
66	Unidentified	*Volutella* ciliata

67	*Xylariales* sp.
68	*Zygosaccharomyces* rouxii
69	Unidentified

a. The numbers indicate the amount of taxonomy detected in current study at each level. The taxonomy information was obtained with higher than 80% confidence.

b. There was not affiliation relationship of the taxonomy in each row.
